# Canine Rabies Ecology in Southern Africa

**DOI:** 10.3201/eid1109.050172

**Published:** 2005-09

**Authors:** John Bingham

**Affiliations:** * Commonwealth Scientific and Industrial Research Organisation (CSIRO) Australian Animal Health Laboratory, Geelong, Victoria, Australia

**Keywords:** rabies, maintenance, persistence, Africa, domestic dog, jackal, metapopulation, perspective

## Abstract

Rabies is a widespread disease in African domestic dogs and certain wild canine populations. Canine rabies became established in Africa during the 20th century, coinciding with ecologic changes that favored its emergence in canids. I present a conceptual and terminologic framework for understanding rabies ecology in African canids. The framework is underpinned by 2 distinct concepts: maintenance and persistence. Maintenance encompasses the notion of indefinite transmission of infection within a local population and depends on an average transmission ratio >1. Maintenance in all local populations is inherently unstable, and the disease frequently becomes extinct. Persistence, the notion of long-term continuity, depends on the presence of rabies in >1 local population within the canine metapopulation at any time. The implications for understanding rabies ecology and control are reviewed, as are previous studies on rabies ecology in African canids.

## Abstract 2

Understanding the persistence of rabies in multiple canine hosts in southern Africa requires applying the principles of metapopulation biology.

The ecologic persistence of pathogenic viruses has been the focus of many studies (*1**–**4*). Rabies virus, a lyssavirus that causes a lethal neurotropic infection of mammals, is a pathogen for which ecologic persistence cannot be explained adequately by pathogenetic mechanisms. Death of the host implies that the virus depends on transmission to new susceptible hosts to survive. However, epidemics, a frequent manifestation of rabies, deplete the number of susceptible hosts, which leads to the decline or extinction of the virus in the affected population. How, then, does the virus persist?

In southern Africa, rabies virus affects many host species, but rabies cycles are sustained by carnivore hosts (*5**,**6*), particularly by canine species (family Canidae), which are the focus of this paper, and by mongooses (family *Herpestidae*) (*5**,**7*), which will not be considered here. Domestic dogs (*Canis familiaris*) are hosts of rabies virus in most of Africa; they cause most human rabies cases and contacts that require medical intervention. In southern Africa, jackals (*C. adustus*, *C. mesomelas*) are also hosts, although their role has been controversial; some studies indicate that they can support rabies cycles (*8*), and other studies indicate that they cannot maintain rabies independently of the disease cycle in dogs (*9**–**12*). Although rabies is a prominent disease of African canids, the mechanisms and hosts responsible for sustaining it have not been clearly elucidated.

I review the ecology of canine rabies in southern Africa, particularly with the goal of resolving the controversies on mechanisms of persistence. A conceptual and terminologic framework to understand the long-term ecologic survival of rabies virus in African canine hosts is proposed.

## Rabies Virus Biology

Rabies virus is transmitted in saliva through the bite of an infected animal. After gaining entry to the central nervous system by peripheral nerves, it causes encephalitis, leading to fulminant, progressive neurologic disease, characterized by excitement, muscular paralysis, impaired responses to social and environmental signals, and other abnormal neurologic signs. The incubation period is unusually variable and can be long; clinical disease and virus shedding are not seen during this period. Infection of the salivary glands during the clinical stage leads to shedding of virions in saliva (*13*).

Rabies virus has a broad mammalian host range. However, in any ecologic zone, a single species, the maintenance host, is usually principally responsible for supporting the virus cycle. The virus variant of the maintenance host is intimately adapted to the host’s physiology and biochemistry to ensure effective transmission (*14*). Maintenance hosts are usually extremely sensitive to their variant but relatively resistant to rabies virus variants of other species. In maintenance hosts, the probability is high that the virus will establish infection, will induce the appropriate behavioral changes, such as aggressive biting behavior, and will prolong the clinical survival period in which salivary virus shedding takes place; all of these factors lead to maximal virus transmission (*14*).

For a virus cycle to be successfully maintained, the average transmission ratio (the average number of new cases caused by each infected host) must be >1. At the beginning of an epidemic, this number is expressed mathematically as the basic reproductive number, *R_0_*, which is defined as the number of new infections generated from an existing infection, when that infection is introduced into a population composed entirely of susceptible hosts (*3**,**15*). *R_0_* is usually treated as a constant that only applies at the beginning of an epidemic, when the ratio of susceptible to infected hosts is at a maximum. *R_0_* will not be used in this article, as it precludes variability in space and time (*16**,**17*); the term average transmission ratio, as defined above, will generally be used instead.

Individuals of species other than the maintenance host may also become infected; they are usually dead-end hosts because of low transmission ratios, which are caused by factors such as the failure to induce biting behavior, inefficient salivary shedding, and absence of other hosts with which to interact. Occasionally, nonmaintenance hosts successfully transmit the infection to conspecifics, which may lead to the establishment of a new cycle if conditions for continual, effective transmission to conspecifics are favorable. The emergence of a new cycle requires some genetic adaptation of the virus in the new host. Lyssaviruses can probably adapt with relative ease because their broad host range allows adaptive selection to take place, as evidenced by the emergence of many new cycles in the last 100 years. A mechanism to explain how such adaptation may arise is the quasispecies concept, where the inherently high mutation rates of RNA viruses produce variant populations of viruses through which selection can act (*18*).

## Definitions and Concepts

Two working definitions will be cited. A local population is a "set of individuals that live in the same habitat patch and therefore interact with each other" (*19*). A metapopulation is a "set of [discrete] local populations within some larger area, where typically migration from one local population to at least some other patches is possible" (*19*). The demographic trends of local populations are asynchronous, particularly where migration between them is relatively low. In this article, the definitions of local population and metapopulation may apply to either the host or the virus. To use an analogy borrowed from ecology, the host local population may be viewed as a resource patch for the pathogen.

Maintenance is the notion of indefinite transmission of virus through members of a host population. (In this context "indefinite" transmission does not mean "permanent" but rather denotes an open-ended cycle that is dependent on availability of susceptible hosts.) The average transmission ratio must be >1 for virus maintenance to be successful. A maintenance host is a member of a population of susceptible individuals that can replicate, shed, and transmit virus efficiently to conspecifics. Maintenance hosts live in local populations, which support indefinite transmission of virus independently of other local populations.

Individual local populations are unlikely to maintain rabies continuously because of the inherently high instability of the disease in any single local population; the disease is normally reintroduced from other infected local populations. Persistence encompasses the concept of long-term and continuous presence of disease within a metapopulation. Successful persistence requires that virus is maintained in >1 local population at any time.

Many viral pathogens depend on a constant supply of susceptible hosts because the viral infection causes either host death or durable immunity. The unstable pattern caused by depletion and renewal of host local populations is a prominent feature of all carnivore rabies cycles, particularly when studied at a relatively fine geographic resolution (*6**,**8**,**20**–**24*).

Mathematical models have shown the importance of features such as host population heterogeneity and mixing. If a spatially heterogeneous host metapopulation experiences a degree of movement between local populations, a pathogen can persist over the long term even though it may frequently become extinct in local populations (*4**,**25**–**28*). Metapopulation heterogeneity may be in terms of density, demographic structure, social interactions, and other characteristics that influence transmission ratios.

Maintenance and persistence of rabies are affected by population immunity, which in effect lowers the average transmission ratio. In carnivores, population immunity against rabies is almost exclusively caused by vaccination rather than natural infection, which is usually fatal.

## Canine Rabies in Southern Africa

In addition to the domestic dog, 3 wild canids have been implicated as independent maintenance hosts of rabies in southern Africa: the side-striped jackal (*C. adustus*), the black-backed jackal (*C. mesomelas*), and the bat-eared fox (*Otocyon megalotis*) (*5**,**6**,**8**,**29*). Rabies cases have also been reported in other African canids, such as African wild dogs (*Lycaon pictus*) (*30*) and Ethiopian wolves (*C. simensis*) (*31*), but these species do not appear to support extended virus cycles independent of other hosts.

In Africa, dogs are intimately dependent on humans for food and shelter (*32**,**33*), and this association means that dog populations can be correlated, in size as well as distribution, with human populations. During the 20th century, the human population of Africa expanded enormously, and the dog population expanded in parallel (*33*). Social changes, such as urbanization, resulted in an increase in human and dog movement. Rabies persistence would have been enhanced in such a dog metapopulation consisting of more numerous local populations and greater movement of infected dogs.

Such a prediction is borne out by historical records. Rabies in sub-Saharan Africa is a disease of modern times; no firm evidence exists of its occurrence before the late 19th century. The first confirmed outbreaks, in South Africa in 1893 and Southern Rhodesia (now called Zimbabwe) in 1902, were in domestic dogs, and their origin was traced to distant lands (*5*). The initial outbreaks were temporally and spatially sporadic, and rabies apparently could not become established. These outbreaks were followed by increasingly frequent, but initially sporadic, outbreaks, until the disease was continuously present in national records, as can be seen in the annual reports of the departments of animal health of various African countries from 1892 to 1960.

Rabies in jackal species appeared in southern and eastern African countries after the introduction of the disease in dogs. In Zimbabwean *C. adustus* populations, rabies occurred in large, dense, moving epidemics in commercial farming areas (*8*). The jackal index cases of the epidemics were usually associated with cases in dogs, which indicates that these epidemics were initiated by dog rabies cycles; once initiated, however, the epidemics were maintained independent of dogs. The moving epidemics terminated at the geographic limits of the *C. adustus* population dominance.

Both jackal species reach high densities in commercial farming environments. Jackal rabies occurs predominantly in these areas, but it is virtually absent from most national parks, despite substantial jackal populations (*8*). Commercial farming practices appear to provide ecologic conditions that are highly favorable for jackals and jackal rabies. What these conditions are is unclear, but they may include abundant resources, increased demographic turnover, and the absence of competitors such as dogs and wild scavengers.

In southern Africa, rabies in *C. mesomelas* and *O. megalotis* predominantly occurs in the absence of domestic dogs or rabies in other carnivores. Given the general lack of associated rabies cases in other species, *C. mesomelas* and *O. megalotis* are likely maintenance host populations, capable of maintaining the virus cycle independent of other species. In *C. mesomelas* and *Otocyon* populations, the mechanisms of rabies maintenance are poorly understood; because surveillance is scanty, discerning true spatial and temporal disease patterns is difficult (*5**,**8**,**29*).

Molecular epidemiology studies have indicated that, in Zimbabwe and South Africa, rabies viruses from dogs and jackals are phylogenetically similar and do not fall into host-distinguishable lineages, while viruses of *O. megalotis* are closely related but distinct (*34*). In addition, viruses all fall within the cosmopolitan lineage that includes many other dog and wild canine virus variants from other regions of the world (*35*). This finding supports the epidemiologic observations that all present-day dog, jackal, and *O. megalotis* rabies viruses in southern Africa stem from a single, recently introduced virus derived from domestic dogs. However, molecular techniques are not precise enough to indicate which species, dogs or the 3 wildlife species, maintains the virus cycles that are found in wildlife. Epidemiologic evidence is required to determine this (*5**,**8**,**29*).

## Review of African Canine Rabies Studies

I propose that African canine rabies ecology must be understood through the distinct concepts of maintenance and persistence. An alternative mechanism proposed for rabies persistence in African domestic dogs includes an infectious healthy carrier status (*10**,**36*). One study proposes this mechanism for rabies in spotted hyenas (*Crocuta crocuta*, Carnivora: *Hyaenidae*), on the basis of rabies virus RNA detected in saliva of healthy hyenas by polymerase chain reaction, although virus isolation, arguably the more important test, was unsuccessful from these samples (*37*). A second mechanism by which rabies may persist is the concept of long incubators, which carry infection through quiescent periods to restart epidemics when the host density has recovered. Long incubators have been reported mainly in humans and other nonmaintenance species (*38**,**39*). No evidence currently shows that carrier animals or long incubators play a role in the persistence of rabies cycles in canine hosts, perhaps because they do not occur frequently enough to be obvious. Although they should not be ignored, these concepts are difficult to demonstrate scientifically and even more difficult to quantify in terms of their ecologic importance for virus persistence.

Previous studies have questioned the ability of jackals to support rabies virus cycles (*9**–**11*). These studies have not distinguished between the ability of species to support pathogen cycles (i.e., maintenance) and the concept of long-term persistence. Acknowledging this distinction would show that local populations of canine species may maintain epidemics independently and may be free of rabies for periods, often long periods, between epidemics. At the level of the local population, this pattern is essentially similar in domestic dogs, jackals, and other canids (e.g., African wild dogs [30]). Domestic dogs may appear to support rabies infection endemically, whereas jackals do not (*11*), simply because more numerous discrete local dog populations are within the study area than are jackal populations.

Considering rabies ecology through the concepts presented here would clarify some of the confusion created by these studies (*9**–**11*). For example, a study (*11*) that used a mathematical model concluded that "the side-striped jackal population itself does not seem able to support rabies infection endemically, i.e., without frequent reintroduction from outside sources of infection." While reintroductions of infection are certainly an important feature in *C. adustus* rabies, reintroduction is probably infrequent, given that most *C. adustus* cases reported in 46 years followed 5 dog-to-jackal initiation events (*8*). The contradiction is resolved once the concepts of maintenance and persistence are applied. Hence, *C. adustus* is capable of maintaining rabies cycles independent of other species, but rabies cycles have not been persistent.

Terms such as reservoir and endemic do not provide the conceptual clarity necessary to understand rabies ecology (*10**,**11*). In a study that acknowledges the complexities of defining the term and uses canine rabies as an example, Haydon et al. (*12*) state that a reservoir is a population "in which the pathogen can be permanently maintained." However, this definition is problematic because the term "permanently maintained" is ambiguous. This study implies that dogs, but not jackals, are reservoirs because they are permanent hosts, yet many dog populations, as with many jackal populations, do not permanently support rabies cycles. Such definitions fail to provide a convincing argument that essential distinctions exist between dogs and jackals in their ability to support rabies cycles. The study by Haydon et al. also defines a reservoir in relation to a target population, which is "the population of concern or interest to us" and which requires protection. Such anthropocentric definitions of pathogen behavior, although having some conceptual value for protecting human health and interests, are unhelpful for understanding the biologic mechanisms of pathogen emergence and persistence.

The scale at which ecologic systems are examined has implications, since disease frequency is less stable in local populations than in metapopulations (*40*). Hence, a farmer will perceive rabies on his property as epidemic in nature, with intense outbreaks separated by long periods of absence, while a national epidemiologist may claim that the disease is endemic in his country. Both observations are correct, but observers perceive the epidemiology differently because they view the disease frequency at different scales. The lump analysis of national rabies case data of Zimbabwe (*10**,**11*) led to a blending and masking of disease patterns, giving these researchers the erroneous impression that rabies frequency in dogs was more stable than it was in jackals.

While the transmission ratio, as influenced by host density, for example, is the only determinant of maintenance, it is not the sole determinant of persistence. In discussing rabies in Zimbabwean jackal populations, Rhodes et al. (*11*) suggest that the "average jackal population density is too low to maintain the chain of infection." However, the dense moving epidemics, which lasted several years and occurred in the absence of cases in other species (*8*), imply that host density was not a limiting factor in jackal epidemics. Once again, we can resolve this apparent contradiction by considering these populations of jackals to be capable of cycle maintenance but not persistence. Transmission is efficient within local populations, but high transmission ratios are transient. The jackal populations of Zimbabwe and the rabies viruses they support should not be considered metapopulations because they do not have, as dog populations do, the spatial separation or interpopulation migration that would be necessary for them to be considered metapopulations. This absence of a metapopulation structure explains the failure of rabies persistence in jackal populations, and this absence, rather than their inability to maintain virus, distinguishes jackals from dogs as hosts of rabies.

## Control of Rabies in Africa

Applying the metapopulation principle to canine rabies in Africa reinforces conventional principles of control: employing vaccination or culling in affected host populations and minimizing movement of infected hosts. For many decades, controlling rabies in Africa, has been the mandate of governments. Vaccination programs for dogs generally consisted of periodic visits by a government vaccination team to communities, which were seen as compliant recipients. While in some cases such government-initiated control efforts arguably had some effect on reducing rabies, they did not cause any long-term trend in reducing rabies in maintenance hosts or humans. African governments have been unable to sustain the level of resource commitment needed to maintain effective levels of vaccination coverage. The metapopulation principle indicates that with increasing dog population density, size, and movement, rabies control will require ever-increasing resources. Traditional methods that have not worked well in the past are likely to be even less effective in the future. Instead, a completely different approach to controlling rabies is needed. Perhaps this approach should be based on community-driven initiatives, where the role of governments focuses on support activities such as surveillance, information dissemination, and legislation. Since dogs are an integral and dependent part of human communities, community-driven initiatives for rabies control may be more sustainable than those directed by governments.

## Conclusions

In recent decades, the frequency of rabies has increased in Africa. Controlling this disease will require a deeper understanding of its biology. When interpreting rabies case data for epidemiologic analysis, we must distinguish between the concepts of maintenance (the ability of local populations to support a disease cycle) and persistence (the presence of >1 infected local population in a host metapopulation). To clearly conceptualize the ecology of canine rabies, we must use lucid, appropriate definitions for virus-host interactions and epidemiologic patterns.

The ecology of many ecosystems has changed dramatically in recent centuries because of the increase in human populations, the introduction of large-scale commercial agriculture, urbanization, loss of biodiversity within the human biosphere, and other changes. The new ecologic landscapes have been exploited by species that can adapt favorably to them, including many of the prominent rabies maintenance hosts. Rabies viruses have recently become prominent in the African ecosystem because of transmission in mammals that have exploited ecologic changes that have occurred in much of the continent. Such change is set to continue into the future, and those species that can flourish under the new conditions will be candidate hosts for the maintenance of pathogens.
